# Psychological resilience as emotional armour in family dysfunction and food addiction among Ghanaian tertiary students

**DOI:** 10.1007/s40519-025-01775-8

**Published:** 2025-08-26

**Authors:** Inuusah Mahama, Christina Ammah, Elizabeth Kwartemaa, Regine Kwaw, Delight Abla Klutsey

**Affiliations:** 1 Department of Counselling Psychology, Faculty of Applied Behavioural Sciences in Education, University of Education, Winneba, Ghana; 2 Department of Educational Foundations, School of Education and Lifelong Learning, University of Education, Winneba, Ghana; 3 Department of Education, Enchi College of Education, University of Ghana, Accra, Ghana

**Keywords:** Psychological resilience, Family dysfunction, Food addiction, Tertiary students, Stress-buffering hypothesis, Ghana

## Abstract

**Objective:**

This study examines the prevalence of family dysfunction and food addiction among tertiary students in Ghana and investigates the moderating role of psychological resilience in this relationship.

**Background:**

Food addiction is an emerging behavioural health concern among tertiary students, particularly in environments characterised by family dysfunction.

**Methods:**

A cross-sectional survey was conducted among a random sample of 553 tertiary students at the University of Education, Winneba. Pearson Product-Moment correlation and the Hayes Process Macro were employed to examine relationships among the study variables and test for moderation effects.

**Results:**

The study found that 26.9% of students experienced high family dysfunction, 39.0% had food addiction, 31.3% were overeaters, and 40.0% had low psychological resilience. Correlation analysis showed weak to moderate positive associations between resilience, family dysfunction, and food addiction. Psychological resilience also buffered the relationship between family dysfunction and food addiction.

**Conclusion:**

The study underscores the interplay between family dysfunction, psychological resilience, and food addiction among tertiary students.

**Implication:**

Universities should implement mental health support services, resilience training programmes, and nutritional education to address food addiction risks among students.

**Level of evidence:**

This study contributes cross-sectional data from Ghana showing that psychological resilience may mitigate the negative effects of family dysfunction on food addiction, providing culturally grounded insight into protective factors in tertiary education settings.

## Introduction

The interplay between family dysfunction and food addiction is a critical yet often overlooked dimension of addictive behaviours and there is scarcity of empirical research on the significance of food addiction among youth [1]. Traditionally, addiction research has centred on substance use disorders [2, 3], yet a growing body of literature indicates that compulsive eating exhibits neurobiological and behavioural parallels with drug addiction [4]. These include shared mechanisms involving reward pathways and impaired executive functioning.

Food addiction is becoming hard to ignore on college campuses. Pursey and colleagues observed that about 24% of students display signs of compulsive eating, a number that outstrips the 20% seen in the wider public [5]. A recent study labelled students a high-risk group because moving away from parental meal routines often upends a young person eating rules [6]. A prior study reported that 15.9% of their sample showed warning signs, reporting on average 3.21 plus-or-minus 1.62 symptoms each [7]. Cebioğlu and colleagues recorded an overall rate of 15.3%, again finding higher figures among men, heavier individuals, and smokers. Together, these numbers point to a pressing public-health worry [8]. Kalon and colleagues also tracked tweaking dopamine signals and broken reward wiring in the brains of people with addictions, zooming in on the amygdala, insula, and nucleus accumbens [9]. Other teams spotted shrinkage in self-control hubs, especially the prefrontal cortex, hinting at shaky impulse steering and decision-making [10, 11]. Heavily processed snacks loaded with extra sugar, fat, and salt fire these circuits hard, triggering what scientists label conditioned hyper-eating.

Some researchers argue that food addiction is more than a brain problem; it also drags in immune, metabolic, and gut glitches [11]. Framed as a behaviour-based addiction with a body component, food addiction mirrors drug-use disorders through mesolimbic dopamine surges, compulsive behaviour, and weakened reward control [12–14]. 

Family dynamics significantly shape the development and maintenance of addictive behaviours, including food addiction. Dysfunctional environments are characterised by emotional neglect, conflict, or inconsistent caregiving and are well-documented risk factors [15–17]. Adverse childhood experiences (ACEs), such as parental substance abuse and emotional neglect, are linked to maladaptive coping strategies. Research shows that insecure attachment and emotion dysregulation mediated the link between ACEs and depression [18]. Similarly, parental emotional availability and discipline have been identified as mediators in the relationship between parental ACEs and children's behavioural outcomes [19].

Empirical studies confirm these findings across diverse contexts. Liu et al. (2023) observed that maladaptive coping strategies and dysfunctional family relationships predicted higher anxiety and depression among college students [20]. Moreover, communication breakdown, lack of emotional expression, and distrust in family relationships were equally common among students [21]. Even in seemingly functional families, it has been reported that ineffective coping mechanisms persisted in over one-third of households, heightening mental health vulnerabilities [22]. 

In such contexts, food often becomes a compensatory mechanism for emotional regulation [23, 24]. Psychosocial models posit that children internalise maladaptive behaviours observed in dysfunctional family systems, including emotional eating and compulsive consumption [25–27]. Parental modelling of disordered eating or addiction further entrenches intergenerational cycles of maladaptive coping [28, 29].

Affective neglect is marked by a lack of validation and emotional support and further exacerbates vulnerability to food addiction [30, 31]. Inconsistent caregiving or disengaged parenting often drives children toward calorie-dense foods that activate reward circuits similar to those engaged by psychoactive substances [32]. Compulsive eating thus emerges as a coping strategy embedded within one’s developmental context, rather than mere behavioural excess.

Psychological resilience is the ability to adapt positively to adversity and is strongly influenced by early familial environments [33, 34]. Studies have shown varying levels of resilience among college students. For example, Al Omari et al. (2023) found that 45.3% of students had low resilience [35]. In contrast, other studies reported high resilience levels among Ghanaian and Middle Eastern student populations, respectively [36, 37].

While resilience is partly intrinsic, it also emerges from environmental factors, such as supportive relationships and positive role models [38–40]. While some scholars contend that family dysfunction can impede resilience development [41, 42], while others highlight that children in adverse environments often cultivate compensatory strategies such as cognitive reframing and problem-solving [43, 44]. Bandura’s theory of self-efficacy also suggests that structured autonomy and problem-solving opportunities can enhance personal competence and resilience [45].

However, resilience can be double-edged. “Survival resilience” may allow individuals to function despite adversity, but at the cost of emotional suppression and hyper-independence—factors that may lead to interpersonal difficulties in adulthood [46, 47]. 

Resilience may also play a protective role by moderating the link between family dysfunction and food addiction. Although most research treats resilience as a mediator [48–54], emerging evidence points to its moderating effects. For example, some studies have found that resilience weakened the association between stress and binge eating, suggesting that resilience served a buffering effect in the relationship [55–57]. 

The current study positions psychological resilience as a moderator in the relationship between family dysfunction and food addiction. This conceptual framework integrates three key constructs: family dysfunction, food addiction, and resilience within a moderation model. Such an approach addresses an underexplored area of research and aligns with calls to identify factors that differentiate at-risk individuals from those who develop adaptive coping mechanisms [58, 59]. 

In sum, while family dysfunction increases vulnerability to food addiction, not all individuals exposed to adverse family environments develop maladaptive behaviours. This discrepancy underscores the importance of psychological resilience as a potential protective factor. By advancing a moderation model, the present study seeks to offer nuanced insights into the mechanisms underlying food addiction among university students. Again, a host of studies discussed psychological resilience in relation to other variables. However, it is clear that studies targeting psychological resilience moderating family dysfunction and food addiction are limited, hence the current study.

## Theoretical framework

This study is grounded in Bronfenbrenner’s Ecological Systems Theory (EST), which posits that human development and behaviour are shaped by dynamic interactions between individuals and their multiple environmental contexts. At the core of the theory is the microsystem, which includes immediate environments such as the family. According to Bronfenbrenner, the quality of family relationships significantly influences developmental outcomes, including behavioural regulation and coping styles [60]. In dysfunctional family environments, it is characterised by poor communication, neglect, or emotional unavailability, where individuals may be more likely to develop maladaptive coping behaviours such as compulsive eating.

The mesosystem reflects the interrelations among microsystems, such as how family experiences influence academic life and peer relationships. For tertiary students, unresolved family conflict may compound academic stress, increasing vulnerability to psychological distress and disordered eating patterns. The exosystem includes broader systems that indirectly affect the individual, such as the availability and quality of university counselling services, while the macrosystem encompasses cultural values and socio-economic conditions that shape coping norms, food availability, and help-seeking behaviours.

By incorporating psychological resilience as a moderator, this study acknowledges that individuals are not passive recipients of environmental stressors. Instead, they actively adapt, and resilience functions as a protective factor that can buffer the adverse effects of family dysfunction. This is consistent with the ecological view that development results from the ongoing, reciprocal interactions between individuals and their environments [61, 62]. 

## The Ghanaian context

Food addiction has emerged as a significant behavioural health concern among tertiary students. Unlike conventional substance use disorders, food addiction is often overlooked due to its social acceptability and the blurred line between normal and pathological eating behaviours. In tertiary students from dysfunctional family backgrounds, food addiction may serve as a maladaptive coping strategy, driven by chronic stress, emotional distress, and impaired self-regulation [5, 63, 64]. 

Dysfunctional family environments create an unpredictable emotional climate, predisposing individuals to heightened psychological stress, which in turn disrupts reward sensitivity and self-regulatory processes. For students who experience such familial instability, food may serve as an emotional regulator, providing transient relief from distress but reinforcing a compulsive cycle of overconsumption [65–67]. In such situations, psychological resilience is often assumed to be a protective factor against maladaptive behaviours like food addiction [68]. However, emerging evidence suggests that resilience may function not only as a buffer but also as an emotional armour, allowing individuals to outwardly withstand adversity while internally maintaining unhealthy coping mechanisms [69]. In the case of food addiction, resilience may prevent overt psychological distress and also enable individuals to sustain addictive eating behaviours [70].

In the Ghanaian context, tertiary students face unique stressors, including academic pressures, financial constraints, and the transition to independent living. For those with a history of family dysfunction, these stressors may exacerbate food addiction risk. Importantly, the interplay between family dysfunction and food addiction in Ghanaian tertiary students remains an underexplored area, particularly in the context of psychological resilience as a potential moderating factor [71, 72]. Therefore, this study seeks to examine the complex interplay between family dysfunction, food addiction, and psychological resilience among tertiary students in Ghana. The study was guided by five questions:

1. What is the level of family dysfunction among tertiary students in Ghana?
2. How prevalent is food addiction among tertiary students in Ghana?
3. What is the level of psychological resilience among tertiary students in Ghana?
4. What is the relationship among family dysfunction, food addiction, and psychological resilience?
5. Does psychological resilience reduce the likelihood of food addiction from family dysfunction?

## Methods

### Study design and participants

The study employed a cross-sectional survey design to recruit 553 (male = 50.1%, female = 49.9%) tertiary students in the university of education, Winneba. In order to achieve wide representation, the study employed a systematic sampling design to select participants from the population of registered undergraduate students using an enrolment list from the university's students' records office. In this case, every 27th student was selected as a participant. The value of 27th was based on the university being made up of 15,231 undergraduate students prior to this sample, and the 553 students needed for the study. Thus, systematic sampling allowed equity of representation, and thus reduced any potential bias that accrues from a convenience sample. The students were basically pursuing programmes in several fields. The mean age of the students was ± 30.36 with a standard deviation of ± 4.90 (between 19 and 52 years), indicating that a significant portion of the sample were mature students. This demographic pattern is acknowledged as a limitation of the study, as it may affect the generalisability of the findings to younger tertiary student populations who might differ in psychosocial maturity, coping mechanisms, and food-related behaviours. Data collection was done at one point in time, between January 2024 and September 2024. This procedure of data collection reflected the precincts of cross-sectional studies [73, 74].

### Study measures

The study adapted three standardised scales to measure family dysfunction, food addiction, and psychological resilience.

### Family dysfunction

The Mcmaster Family Assessment Device [FAD] short version with 12-items [75] was used to assess family dysfunction among tertiary students. The scale had some statements negatively worded (2, 4, 6, 8, 12), which were reverse scored to avoid interpretive bias. The scale was scored on a four-point Likert, from strongly agree denoting 4 to strongly disagree denoting 1. The FAD items were reviewed by experts in family psychology in Ghana to ensure cultural appropriateness. The wording and meaning of items were carefully examined to maintain their conceptual integrity in the local context. It was further pre-tested among 65 students in a nearby university, it was and deemed to have met the necessary criteria for use in the current situation. A reliability coefficient of 0.77 was produced after psychometric examination.

### Food addiction

The Yale Food Addiction Scale [Modified YFAS] was used to assess food addiction among tertiary students. Specifically, the uni-dimensional briefer version 13-items was used. The scale was scored on a four-point Likert, from strongly agree denoting 4 to strongly disagree denoting 1. The YFAS items were pre-tested among 65 Ghanaian students in a neighbouring university to ensure clarity, cultural sensitivity, and relevance to food-related behaviours in Ghana. Minor linguistic modifications were made where necessary. The scale produced a reliability coefficient of 0.89 after psychometric examination.

### Psychological resilience

The Resilience Evaluation Scale [76] was used to assess psychological resilience among tertiary students. It had nine items distributed between two dimensions: self-confidence and self-efficacy. The scale was scored on a four-point Likert, from strongly agree denoting 4 to strongly disagree denoting 1. Experts in clinical psychology and resilience research reviewed the scale to ensure that the constructs of self-confidence and self-efficacy were culturally appropriate and aligned with how resilience is conceptualised in Ghanaian society. The scale was subjected to pre-testing among 65 students in another university, and it met the criteria for use in this current study. In all, the scale produced a composite Cronbach alpha value of 0.88.

### Analysis procedures

To address research question five, the Hayes PROCESS Macro (Model 1) in SPSS (version 25) was used to assess the moderating effect of psychological resilience on the relationship between family dysfunction and food addiction. Moderation was tested by including an interaction term (family dysfunction × resilience) in the regression model. This approach allowed for the identification of conditional effects, showing whether the impact of family dysfunction on food addiction varied depending on levels of resilience.

### Ethical consideration

Before the commencement of data collection, ethical clearance was granted by the Research Review Committee of the University of Education (UEW/IRB/2024), Winneba. The study protocol was subsequently evaluated and approved. All aspects of the study procedures involving human subjects were conducted in line with the ethical principles set forth in the 2013 Declaration of Helsinki and as articulated within the ethics protocols of the University of Education, Winneba. Participants were provided with information about the research purpose, clearly communicated their rights to confidentiality and participation was clearly voluntary, and they were required to provide written informed consent prior to partaking of the survey.

## Results

From Table 1, the gender distribution of the sample (*n* = 553) is remarkably balanced, with males constituting 50.1% (*n* = 277) and females comprising 49.9% (*n* = 276). This near-equal representation suggests that gender is unlikely to introduce significant bias in the study’s findings, allowing for a more generalisable interpretation across male and female participants. Given the minimal difference between the two groups, any observed gender-related effects are likely to be influenced by factors beyond mere numerical representation, necessitating a more nuanced analysis of potential interactions between gender and the study’s primary variables.

**Table 1:** Gender of respondents

Sex		Frequency	Percent
	Male	277	50.1
	Female	276	49.9
	Total	553	100.0

The descriptive statistics presented in Table 2 provide insights into the central tendencies and distributional properties of the study’s key psychological variables. Psychological resilience (*M* = 33.77, SD = 5.07) exhibits a negative skewness (− 1.34) and a moderately peaked distribution (Kurtosis = 2.99). The negative skew suggests that most participants scored above the mean, indicating a tendency toward higher resilience levels. The kurtosis value approaching 3 suggests a distribution shape similar to the normal curve. Also, family dysfunction (*M* = 35.24, SD = 5.45) is approximately normally distributed, as evidenced by minimal skewness (− 0.17) and kurtosis (− 0.05). These values suggest that family dysfunction scores are symmetrically distributed, with no extreme deviations from normality. Likewise, food addiction (*M* = 71.83, SD = 11.44) also exhibits near-normality, with skewness (0.05) and kurtosis (− 0.11) close to zero suggesting a well-balanced distribution with no substantial clustering of scores at the high or low end. From a clinical and research perspective, the non-normal distribution of psychological resilience warrants further examination, as a negatively skewed distribution could imply that protective factors against stress and adversity are prevalent among the sample. Meanwhile, the relative normality in family dysfunction and food addiction insinuates that these constructs exhibit a more typical variability within the population.

**Table 2:** Descriptive statistics

Variables	*N*	Mean		SD	Skewness		Kurtosis	
	Stat	Stat	S. E	Stat	Stat	S. E	Stat	S. E
Psychological resilience	553	33.77	0.22	5.07	− 1.34	0.10	2.99	0.21
Family dysfunction	553	35.24	0.23	5.45	− 0.17	0.10	− 0.05	0.21
Food addiction	553	71.83	0.49	11.44	0.05	0.10	− 0.11	0.21

## What is the level of family dysfunction among tertiary students in Ghana?

In answering the question, FAD was used and this scale had score range between 1 and 4. A cut-off score of 2.00 is typically used to distinguish between healthy and unhealthy family functioning. So, ≤ 2.00 denotes healthy family functioning while > 2.00 denotes unhealthy family functioning. Table 3 presents the results.

**Table 3:** Family dysfunction

Levels	Frequency	Percent
Low	404	73.1
High	149	26.9
Total	553	100.0

Table 3 provides an overview of family dysfunction levels among tertiary students in Ghana. The majority of students (73.1%, *n* = 404) report low family dysfunction, indicating that a significant portion of the sample experiences relatively stable and functional family environments. In contrast, 26.9% (*n* = 149) of students fall into the high family dysfunction category, indicating a substantial minority facing familial challenges that could potentially impact their psychological well-being and academic performance. From a psychological perspective, these findings underscore the importance of considering family dynamics when assessing student mental health and resilience. While most students report lower dysfunction levels, the one in four students experiencing high family dysfunction represents a notable risk group.

## How prevalent is food addiction among tertiary students?

In answering the question, YFAS rated 4-point was used. With this, symptom count reflects the number of addiction-like criteria endorsed, while diagnosis indicates whether a threshold of three or more symptoms plus clinically significant impairment or distress has been met. Table 4 presents the results.

**Table 4:** Food addiction

Levels	Frequency	Percent
Average eaters	164	29.7
Overeaters	173	31.3
Food addicts	216	39.0
Total	553	100.0

Table 4 presents the prevalence of food addiction among tertiary students. The findings indicate that a substantial proportion of students exhibit problematic eating behaviours, with 39.0% (*n* = 216) classified as food addicts. This suggests that nearly four in ten students demonstrate eating patterns consistent with compulsive food consumption, which may have implications for both physical and mental health. Additionally, 31.3% (*n* = 173) of students fall into the overeaters’ category, highlighting a potential risk for progression into food addiction. meanwhile, only 29.7% (*n* = 164) are classified as average eaters, indicating that less than a third of the sample maintains relatively balanced eating habits. These findings raise important concerns about dietary habits and potential health risks among tertiary students. Given the high prevalence of food addiction (39.0%) and overeating (31.3%), further investigation is warranted to explore underlying psychological factors, such as stress, emotional dysregulation, and coping mechanisms, which may contribute to disordered eating behaviours in this population.

## What is the level of psychological resilience among tertiary students?

The question was answered using the psychological resilience scale scored on 4-point. Based on the distribution of scores, the following thresholds ≤ 2.5 denoting low resilience, 2.6 and 3.5 denoting moderate resilience, and > 3.5 denoting high resilience were applied. Table 5 presents the results.

**Table 5:** Psychological resilience

	Levels	Frequency	Percent
	Low	221	40.0
	Moderate	152	27.5
	High	180	32.5
	Total	553	100.0

Table 5 presents the distribution of psychological resilience levels among tertiary students. The results indicate that 40.0% (*n* = 221) of the students exhibited low psychological resilience, while 27.5% (*n* = 152) demonstrated a moderate level of resilience. A notable 32.5% (*n* = 180) of the students reported high psychological resilience. These findings suggest that a significant proportion of tertiary students’ experience lower levels of resilience, which may have implications for their ability to cope with academic and personal stressors.

## What is the relationship among family dysfunction, food addiction, and psychological resilience?

To address this question, a moderation analysis was conducted using the Hayes PROCESS Macro (Model 1). This analysis aimed to determine whether psychological resilience moderates the relationship between family dysfunction and food addiction among higher education students. The moderation model was specified with family dysfunction as the independent variable, food addiction as the dependent variable, and psychological resilience as the moderator. An interaction term (family dysfunction × psychological resilience) was created within the model to test for conditional effects. This enabled the examination of whether the strength or direction of the relationship between family dysfunction and food addiction varies depending on the level of psychological resilience. Table 6 presents the results.

**Table 6:** Correlations matrix of the study variables

Variables		1	2	3
Psychological resilience (1)	Pearson correlation	1		
	Sig. (2-tailed)			
Family dysfunction (2)	Pearson correlation	0.087^*^	1	
	Sig. (2-tailed)	0.042		
Food addiction (3)	Pearson correlation	0.185^**^	0.303^**^	1
	Sig. (2-tailed)	0.000	0.000	

In Table 6, the preliminary analyses using Pearson’s product-moment correlation provided insight into the relationships among the key variables. A weak but statistically significant positive correlation was observed between psychological resilience and family dysfunction (*r* = 0.087, *p* = 0.042). While the effect size was minimal, the finding suggests a slight association wherein individuals from dysfunctional family backgrounds may develop adaptive resilience mechanisms, potentially as a compensatory response to adversity. However, this interpretation should be made cautiously due to the small magnitude of the effect. Interestingly, a moderate and statistically significant positive correlation was also found between psychological resilience and food addiction (*r* = 0.185, *p* < 0.001). This result is somewhat counterintuitive, as resilience is typically associated with protective effects against maladaptive behaviours. The finding may imply that resilience, in certain contexts, is expressed through coping strategies that inadvertently reinforce compulsive eating behaviours. For instance, individuals may use food to self-soothe while maintaining an outward appearance of functioning, particularly when faced with unresolved emotional stressors. Additionally, family dysfunction and food addiction were moderately correlated (*r* = 0.303, *p* < 0.001), suggesting that individuals who experience higher levels of family dysfunction are more likely to engage in food addiction behaviours. This supports prior literature indicating that dysfunctional family environments can foster maladaptive coping strategies, including disordered eating patterns.

## Does psychological resilience reduce the likelihood of food addiction from family dysfunction?

In Table 7, the results of the moderation analysis revealed that the overall model was statistically significant, *F* (3, 549) = 29.71, *p* < 0.001, with an *R*^2^ value of 0.14. This indicates that the model accounted for approximately 14% of the variance in food addiction scores. Both family dysfunction and psychological resilience emerged as significant predictors of food addiction. Specifically, family dysfunction was positively associated with food addiction (*B* = 3.91, SE = 0.51, *t* = 7.61, *p* < 0.001), indicating that students who reported higher levels of dysfunction within their family systems were more likely to exhibit symptoms of food addiction. Likewise, psychological resilience was also found to be a significant predictor (*B* = 5.66, SE = 1.07, *t* = 5.28, *p* < 0.001), reinforcing the earlier correlational result, though these finding challenges conventional assumptions that resilience solely functions as a buffer against maladaptive behaviour.

**Table 7:** Moderation results for resilience moderating family dysfunction and food addiction

*R*	*R*-square	MSE	*F*	df1	df2	*P*
0.37	0.14	113.18	29.71	3.00	549.00	0.000
**Variable**	**Coeff**	**SE**	**T**	***p***	**LLCI**	**ULCI**
**Constant**	65.96	0.91	72.56	0.000	64.17	67.74
Family dysfunction	3.91	0.51	7.61	0.000	2.90	4.92
Food addiction	5.66	1.07	5.28	0.000	3.56	7.76
Interaction_1	− 2.35	0.62	− 3.78	0.002	− 3.57	− 1.13

Most importantly, the interaction term between family dysfunction and psychological resilience was statistically significant (*B* =  − 2.35, SE = 0.62, *t* =  − 3.78, *p* = 0.002). The negative coefficient for the interaction suggests a moderating effect, where psychological resilience reduces the strength of the relationship between family dysfunction and food addiction. In other words, while family dysfunction is generally associated with higher levels of food addiction, the presence of psychological resilience appears to buffer this effect. This finding aligns with the stress-buffering hypothesis, which posits that internal or external protective factors such as resilience can mitigate the adverse psychological outcomes associated with chronic stress or trauma. Therefore, students with higher resilience scores were less affected by the negative influence of family dysfunction on their eating behaviours, demonstrating that resilience plays a critical role in safeguarding mental and behavioural health in high-risk environments.

**Fig. 1:**
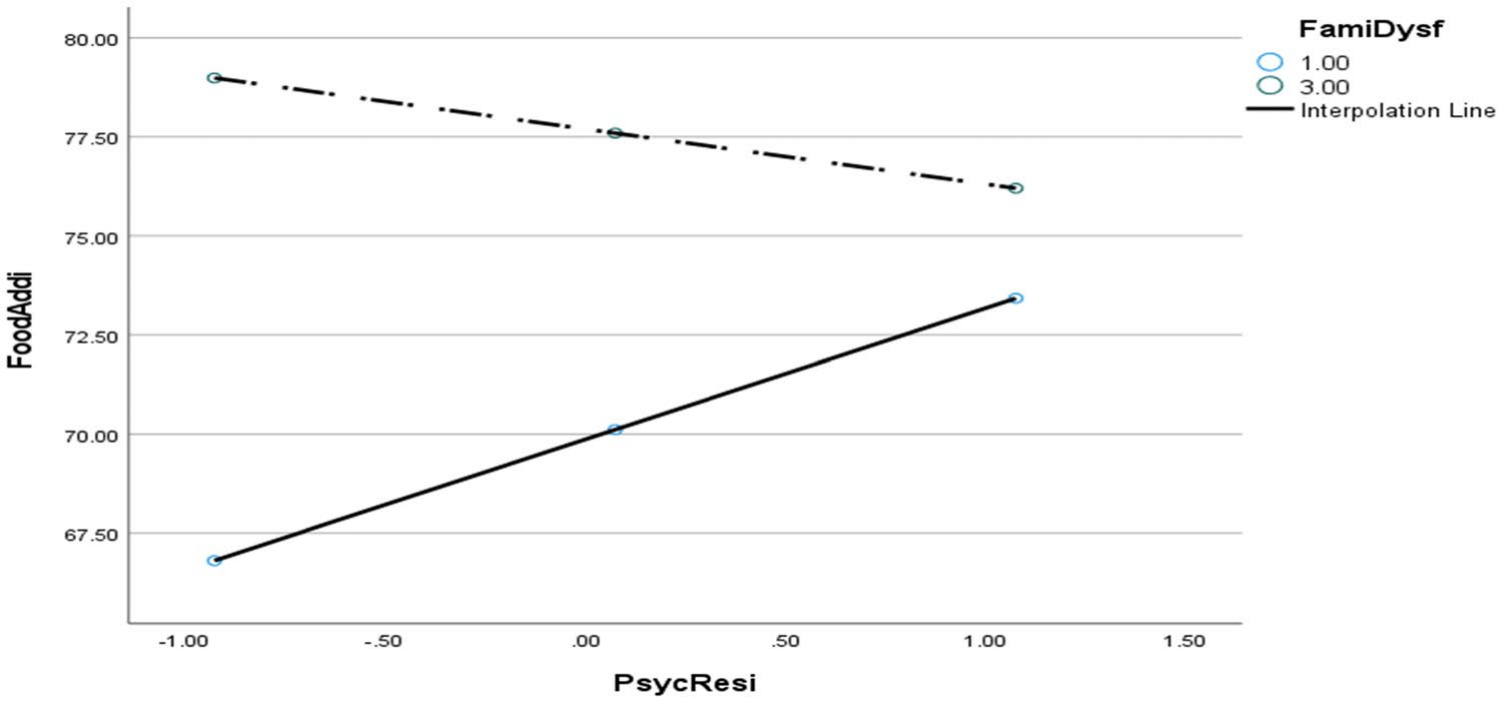
Interpolation graph

Taken together, these findings underscore the complex and sometimes paradoxical nature of psychological resilience in the context of food addiction. While resilience is generally beneficial, it may manifest differently across individuals, particularly those navigating emotionally challenging family systems. The results affirm the need for more nuanced understandings of resilience as both a direct predictor and a moderator, and highlight the importance of fostering adaptive forms of resilience in tertiary education contexts to buffer against the risk of food-related behavioural issues. Figure 1 lends further explanations.

## Discussion of the findings

The present study revealed that a majority of tertiary students in Ghana (73.1%) reported low levels of family dysfunction. This finding suggests that while a substantial proportion of students come from relatively stable family environments, a significant minority face family-related challenges that may affect their psychological and academic adjustment. Although this mirrors previous studies highlighting the influence of family dynamics on student mental health [20, 77], the implication for Ghanaian tertiary education is that interventions aimed at supporting students’ family communication and emotional well-being remain essential. Even within families classified as “functional”, coping difficulties can still manifest, as evidenced in the work of Ahmed Osman et al. (2024), where many students from functional family settings still exhibited psychological distress [22]. This underscores the importance of moving beyond binary labels of “functional” versus “dysfunctional” to understand how nuanced family experiences shape emotional outcomes.

A particularly concerning finding was the high prevalence of food addiction and overeating among the sample. Specifically, 39.0% of participants met the criteria for food addiction, with an additional 31.3% falling into the overeating category. In effect, nearly seven in ten students exhibited maladaptive eating behaviours. This prevalence is markedly higher than that reported in prior studies [7], which identified food addiction rates of 15.9% among university students. While population and methodological differences may account for some of this discrepancy, the elevated rates observed in this study raise serious concerns about long-term health outcomes, including obesity, metabolic disorders, and psychological comorbidities. The findings suggest that the university environment in Ghana may expose students to heightened stressors such as academic pressure, poor dietary choices, and socio-economic constraints that facilitate compulsive eating as a coping mechanism. This pattern invites urgent institutional responses, including mental health screening, nutritional counselling, and campus-wide wellness interventions.

An equally important insight emerged from the resilience data. While 32.5% of students exhibited high psychological resilience, 27.7% and 40.0% demonstrated moderate and low levels, respectively. This distribution suggests that a significant portion of students may lack the psychological tools needed to adapt to challenges effectively. In contrast to Mahama and colleagues, who found high academic resilience among Ghanaian university students, the current findings suggest variation that could be attributed to differing family experiences, educational contexts, or even socio-economic pressures [36]. This variation is further supported by findings from Jawad and Mohammed (2022), whose participants generally reported higher resilience (37). The present results highlight the need for resilience-building strategies that are contextually tailored to the psychosocial realities of Ghanaian students.

Critically, the correlational findings from the study revealed a moderate positive association between psychological resilience and food addiction—a novel and somewhat paradoxical result. Traditionally, resilience is viewed as a protective factor against maladaptive behaviours. However, the data suggest that individuals with higher resilience may also exhibit higher tendencies toward food addiction. This challenges the simplistic view of resilience as universally beneficial. A plausible interpretation is that some students exhibit what researchers’ term *“survival resilience”*—a form of coping marked by high functionality and persistence, but underpinned by emotional suppression or avoidance strategies such as compulsive eating. In such cases, food may serve as a self-regulatory tool to mask emotional pain while preserving outward performance. Resilience is not inherently adaptive unless coupled with healthy emotional regulation [59]. Hence, this finding calls for a more nuanced conceptualisation of resilience—not as a linear predictor of well-being, but as a multidimensional process that can manifest in both protective and maladaptive ways, depending on the psychosocial context.

Furthermore, the study confirmed a moderate positive correlation between family dysfunction and food addiction, reinforcing existing literature that links adverse family environments to disordered eating. More importantly, the moderation analysis revealed that psychological resilience significantly buffered this relationship. The interaction effect showed that the impact of family dysfunction on food addiction was less severe among students with higher resilience. This finding supports the stress-buffering hypothesis, which posits that resilience can attenuate the negative effects of environmental stressors. In practical terms, students who demonstrate higher resilience are less likely to develop compulsive eating behaviours even when exposed to dysfunctional family settings. This reinforces the importance of university-based resilience enhancement programmes, particularly those that include emotional literacy, adaptive coping skills, and community-building initiatives.

## Conclusion

The study underscored the crucial influence of family dynamics on the psychological well-being of tertiary students, with most participants reporting low levels of family dysfunction. However, findings also revealed a high prevalence of food addiction and overeating, indicating that most students exhibited problematic eating behaviours. Additionally, a considerable proportion of students demonstrated low levels of psychological resilience, which may hinder their ability to cope with academic and personal stressors. The study further identified a weak correlation between resilience and family dysfunction, a moderate correlation between psychological resilience and food addiction, and a moderate correlation between family dysfunction and food addiction. Most importantly, psychological resilience was found to moderate the relationship between family dysfunction and food addiction, suggesting that higher resilience levels can buffer the negative impact of family dysfunction on maladaptive eating behaviours.

## Implications for practice and policy

1. Universities should incorporate structured screening for family dysfunction and psychological distress during student orientation or through academic advising frameworks. This can be done using brief, validated tools such as the Family Assessment Device (FAD) and General Health Questionnaire (GHQ-12) to detect at-risk students. Students identified as vulnerable should be referred to university counselling centres for further psychological assessment and intervention. In addition, counselling units should be resourced with trained professionals capable of addressing family-related stress and its behavioural consequences, including disordered eating and emotional dysregulation.
2. While university-based interventions are critical, addressing the root of family dysfunction requires engaging families. Institutions could partner with community and religious leaders to facilitate parenting workshops, targeting emotional communication, attachment styles, and healthy conflict resolution. Such outreach would enhance the family’s capacity to support students' emotional needs and academic adjustment, particularly during transitions to university life.
3. To address the high prevalence of food addiction and overeating, universities should establish comprehensive nutrition awareness programmes. These could include workshops, peer-led seminars, and online modules that promote mindful eating, balanced diets, and awareness of the psychological triggers for compulsive eating. Integrating such programmes into student wellness weeks or health fairs would increase visibility and engagement. Access to healthy meal options on campus should also be expanded.
4. Resilience education should not be limited to counselling centres; it must be embedded within the university’s broader academic and co-curricular offerings. Universities can develop first-year seminars or foundation courses that include modules on emotional intelligence, stress management, self-reflection, and adaptive coping strategies. These programmes should be delivered using experiential learning formats such as journaling, group discussions, and case-based scenarios to encourage self-awareness and skills application.
5. University-based psychologists and trained facilitators should implement structured resilience-building programmes. These programmes can follow evidence-based models such as the Penn Resilience Program (PRP) or the Stress Management and Resilience Training (SMART) model, adapted for local contexts.

### What is already known on this subject?

Previous research has identified associations between family dysfunction and food addiction, but limited evidence exists from sub-Saharan African contexts, particularly among university students.

### What this study adds?

This study contributes cross-sectional data from Ghana showing that psychological resilience may mitigate the negative effects of family dysfunction on food addiction, providing culturally grounded insight into protective factors in tertiary education settings.

## Data Availability

No datasets were generated or analysed during the current study.

## Declarations

### Competing of interest

The authors have no interest to declare in as much as idea conceptualisation and conducting of this paper are concerned.
